# Mechanism of Mutation-Induced Effects on the Catalytic Function of TEV Protease: A Molecular Dynamics Study

**DOI:** 10.3390/molecules29051071

**Published:** 2024-02-29

**Authors:** Jingyao Wang, Yicong Xu, Xujian Wang, Jiahuang Li, Zichun Hua

**Affiliations:** 1 School of Biopharmacy, China Pharmaceutical University, Nanjing 211198, China; 15305188317@163.com (J.W.); minexyc12@163.com (Y.X.); hsuchein0126@outlook.com (X.W.); 2 Changzhou High-Tech Research Institute, Nanjing University, Changzhou 213164, China; 3 State Key Laboratory of Pharmaceutical Biotechnology, School of Life Science, Nanjing 210023, China

**Keywords:** TEV protease, molecular dynamics simulations, catalytic efficiency, mutational mechanistic

## Abstract

Tobacco etch virus protease (TEVp) is wildly exploited for various biotechnological applications. These applications take advantage of TEVp’s ability to cleave specific substrate sequences to study protein function and interactions. A major limitation of this enzyme is its relatively slow catalytic rate. In this study, MD simulations were conducted on TEV enzymes and known highly active mutants (eTEV and uTEV3) to explore the relationship between mutation, conformation, and catalytic function. The results suggest that mutations distant from the active site can influence the substrate-binding pocket through interaction networks. MD analysis of eTEV demonstrates that, by stabilizing the orientation of the substrate at the catalytic site, mutations that appropriately enlarge the substrate-binding pocket will be beneficial for Kcat, enhancing the catalytic efficiency of the enzyme. On the contrary, mutations in uTEV3 reduced the flexibility of the active pocket and increased the hydrogen bonding between the substrate and enzyme, resulting in higher affinity. At the same time, the MD simulation demonstrates that mutations outside of the active site residues could affect the dynamic movement of the binding pocket by altering residue networks and communication pathways, thereby having a profound impact on reactivity. These findings not only provide a molecular mechanistic explanation for the excellent mutants, but also serve as a guiding framework for rational computational design.

## 1. Introduction

With the advances in biotechnology and bioengineering, there have been significant improvements in recombinant protein production, which have lifted proteins from the constraints imposed by natural sources and facilitated the exploration of a diverse array of proteins [1,2]. Tobacco etch virus protease (TEVp) is a widely utilized protease in the field of biotechnology. TEVp exhibits high specificity in recognizing the target sequence ENLYFQG/S and cleaving between the residues Q and G/S [3]. It is an excellent tool for the precise elimination of tags and undesired sequences or to achieve accurate processing of the defined polypeptide, which can be utilized for removing N- or C-tags from recombinant fusion proteins [4,5], conducting in vitro enzymology testing [6], serving as biosensors for monitoring protein–protein interactions (PPIs) within living cells [7], and displaying activity in mammalian cytosol to recognize a seven-amino-acid consensus peptide substrate [8]. 

TEVp belongs to the serine protease family and its structure features two antiparallel β-sheet domains that fold together like a bucket. The catalytic triad residues, including His46, Asp81, and Cys151, are positioned at the intersection of these two domains [4]. The most significant advantage of TEVp is its stringent site recognition specificity, as it has not been observed to cleave fusion proteins at non-specific sites thus far [9,10]. However, the self-cleavage and the relatively low enzyme activity of TEVp limit its application, making rational screening and design of TEVp is essential. To date, there have been few successful cases of rational design of TEVp. In some successful cases of enzyme modification, the method used was directed evolution [11,12,13,14,15,16,17].

Previous studies have shown that the self-cleavage of TEV protease can be avoided while maintaining unchanged enzyme activity by replacing Ser219 with Val [12,13]. Since most commercial proteases possess a Val at this site, in this study we used the TEV protease with V219 as the reference protease (named WT). Based on directed evolution studies, new mutation sites within TEV protease have been identified and investigated recently. Among these mutations, two groups of mutation sites were strategically designed, leading to remarkable improvements in activity as shown in Table 1. Denard et al. [16] described a highly versatile version of the yeast endoplasmic sequestration screening system (YESS 2.0) utilizing both error-prone library and saturation mutagenesis libraries. This allowed them to identify two optimal variants, E2 (S3I, P8Q, S31T, A231V) and S7 (E79G, V219R), which were then combined to form the hexamutant-enhanced TEV (eTEV: S3I, P8Q, S31T, T173A, V219R, A231V). The eTEV variant demonstrated specific digestion of the fusion protein within 2 hours at an enzyme to substrate ratio of 1:200, with a catalytic efficiency 2.25-fold higher than WT. This enhancement was primarily due to a notable increase in its turnover rate (Kcat). In 2020, Mateo et al. [17] also developed a yeast-based platform for directed evolution of protease catalytic properties, resulting in a faster variant of TEV protease (uTEV3) after multiple rounds of selection. The Kcat/Km value of uTEV3 reached 6.82, nearly threefold higher than that of the WT, primarily due to a threefold reduction in Km, rather than an increase in Kcat. These screened high-enzyme-activity mutants may provide a structural and functional basis for rational design of enzyme modifications.

In the present study, we performed molecular dynamics simulations of WT and its two highly active variants (uTEV3 and eTEV) to explore the relationship between mutation, conformation, and catalytic function. By analyzing the structural dynamics and interactions within the active site, we were able to identify mechanisms responsible for the enhanced catalytic activity in TEVp. These findings not only provide a molecular mechanistic explanation for the excellent mutants, but also serve as a guiding framework for rational computational design.

## 2. Results

### 2.1. Overview of TEVp Structures and MD Simulations

**Figure 1:**
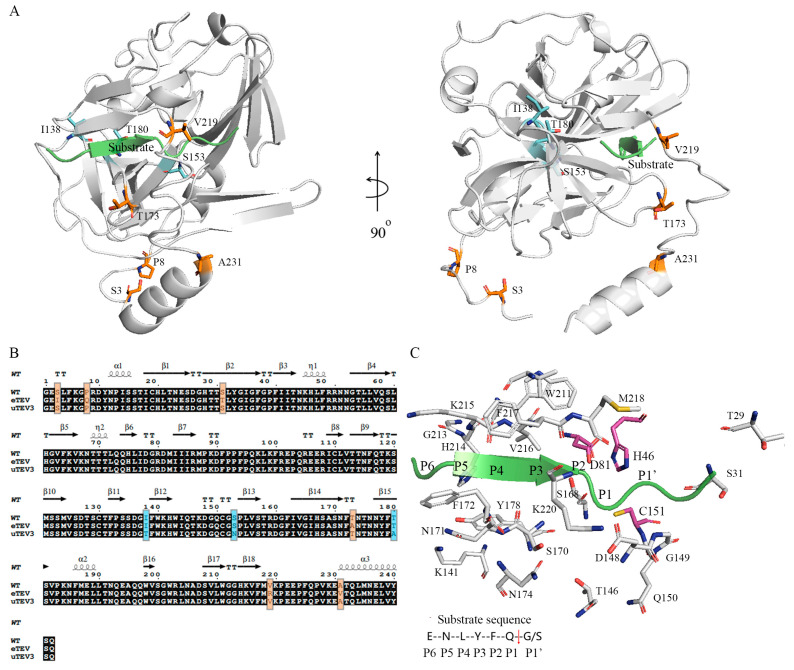
The structural model of full-length wild-type TEV protease. (**A**) The tertiary structure of TEV protease–substrate complex. The mutation sites of the mutants eTEV and uTEV3 are shown in orange and blue, respectively, and the substrate (peptide ENLYFQSG) is shown as a green ribbon. (**B**) Sequence alignment of WT, eTEV, and uTEV3. (**C**) The substrate-binding pocket of TEV protease, containing catalytic triad residues (H46, D81, and C151 shown as purple violet sticks).

The models of full-length wild-type (WT) TEVp–substrate complexes are shown in Figure 1, and the mutation sites of eTEV and uTEV3 are shown in the structure. The binding pocket of TEVp is formed by Thr29, Ser31, His46, Asp81, Thr146, Asp148-Cys151, His167-Phe172, Asn174, Tyr178, Trp211, and Gly213-Lys220. Structural analysis shows that mutant residues in the high-active variants (eTEV and uTEV3) obtained through directed evolution are mostly situated distant from the active site. The comparison of sequences and structures between WT and its two variants cannot explain the different catalytic activities, and the impact of these mutations on enzyme activity is noteworthy. To understand the effect of the mutations on the structure and function of TEVp, we employed MD simulations to investigate the dynamic conformational changes.

**Figure 2:**
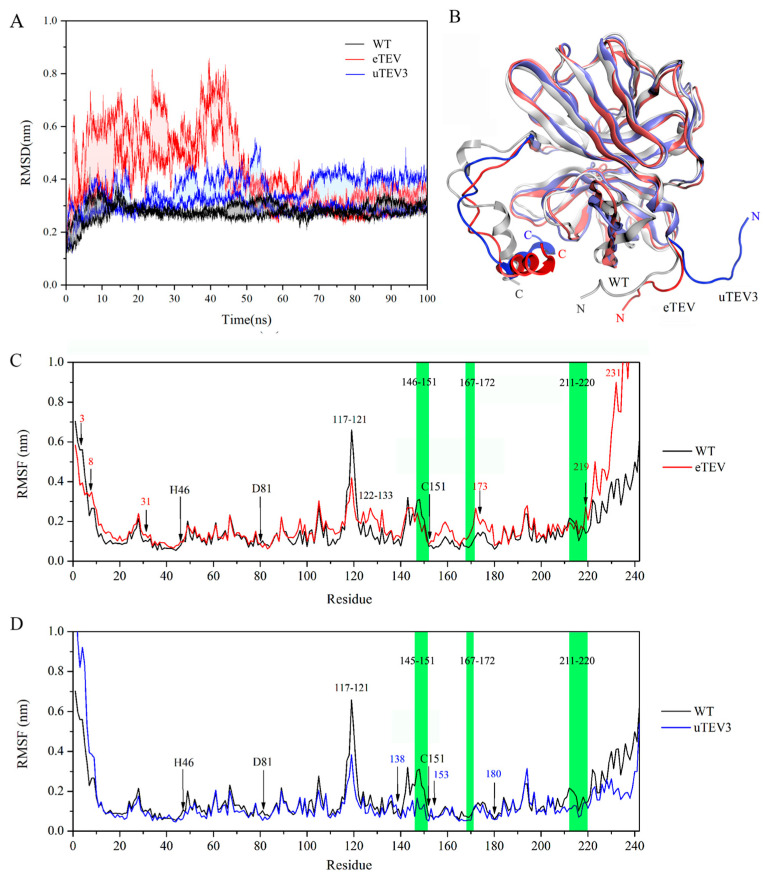
(**A**) The α-C RMSD values of TEV-WT, eTEV, and uTEV3 during the 100 ns dynamic molecular simulation process. The calculation was repeated twice and generated similar results. (**B**) Schematic diagram of structure superposition after 100 ns MD simulation of three systems. (WT represented in gray, eTEV represented in red, and uTEV3 represented in blue.) (**C**,**D**). The root mean square fluctuations (RMSF) of the residue of eTEV and uTEV3 system compared with WT. Mutation site residues are labeled in red (eTEV) and blue (uTEV3), respectively. The green regions of 145–151, 167–172, and 211–220 are substrate-binding sites.

MD simulations on WT, eTEV, and uTEV3 were carried out in our work to obtain the corresponding stable structure. The root mean square deviation (RMSD) of Cα atoms of protein compared to the initial conformation was plotted in Figure 2A. Compared with WT, the structures of eTEV and uTEV3 had undergone significant adjustments. In particular, the eTEV experienced significant changes in RMSD during the first 40 ns. However, after 70 ns, all structures reached a stability, and the converged RMSD with respect to the starting conformation stabilized at 0.30 ± 0.02 nm (WT), 0.28 ± 0.02 nm (eTEV), and 0.41 ± 0.02 nm (uTEV3), respectively. Thus, the simulated trajectories of the final 30ns were chosen for further analysis. The structural comparison showed that the structural changes in the variants mainly occur at the two ends of the protein, especially at the C-terminus (Figure 2B).

To analyze the dynamic changes caused by mutations, we calculated the root mean square fluctuation (RMSF) of the residues during the simulated time (Figure 2C,D). The RMSF plot of eTEV showed that mutations significantly affected the dynamic behavior of the mutation sites and their surrounding regions. Compared to WT, the flexibility of mutated residues 8, 31, 173, 219, and 231 in eTEV was significantly increased, especially the mutations of C-terminal V219R and A231V had a significant impact on the structure of the C-terminal region. In addition, mutations in eTEV led to large RMSF values around residues 167–172 (substrate-binding site), indicating that these mutations might affect the flexibility of the peptide binding pocket. However, the RMSF value of region Thr146-Cys151 appeared to be much lower compared to WT, indicating high stability in the binding region near the catalytic residue Cys151 in variant eTEV (Figure 2C). In contrast to eTEV, the flexibility of mutation sites 138, 153, and 183 in variant uTEV3 were not affected by mutations (Figure 2D). Furthermore, the conformational changes between uTEV3 and WT by RMSF showed that the binding pocket of residues in regions of residues 146–151, 167–172 and 211–220 of the uTEV3 showed smaller fluctuations, which indicated that the stability of the binding pocket might be improved because of mutations. Our results also showed that for both mutants eTEV and uTEV3, catalytic residues His46, Asp81, and Cys151 displayed similar values of RMSF among all enzymes, suggesting the stability of TEVp’s catalytic center.

**Figure 3:**
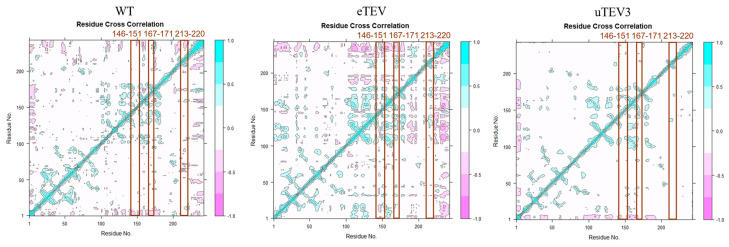
Dynamic cross-correlation map (DCCM). The DCCM map for wild-type, eTEV, and uTEV3 shows the correlated motions of protein residues in wild-type and mutant complexes. The cyan color represents a positive correlation and the pink color represents a negative correlation. The color gradients represent a gradual decrease in the correlation.

**Figure 4:**
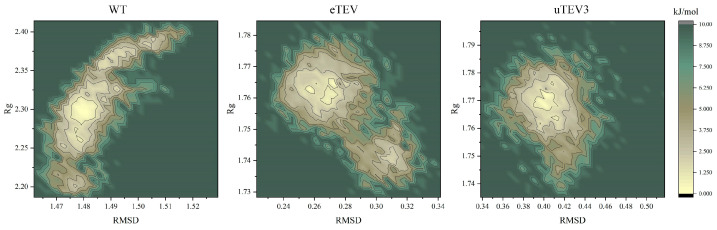
Free-energy landscape maps, where the yellow color area indicates lower energy.

To clarify the mechanism of the flexible regional change and the biological function of mutations, analyses of dynamic cross-correlation maps were carried out and presented in Figure 3. The findings plainly confirmed that due to mutations, the distinct interatomic motion covariance pattern of the substrate-binding pocket was significantly changed, and different patterns of correlated motions were observed in WT, eTEV, and uTEV3 systems. In eTEV, the correlated motion between His167 and Thr171 was significantly increased, indicating a significant change in the interaction between residues after mutations. However, in uTEV3, the negative correlation motion between Gly213 and Lys220 was significantly attenuated (Figure 4), suggesting that the mutations led to changes in the original related motions between amino acid/residues and then affected the conformation of the binding pocket.

Changes in free-energy landscapes caused by mutations were presented in Figure 4. Free-energy landscape maps (FELs) can efficiently evaluate enzyme activity by identifying the conformational changes in protein in dynamic equilibrium. In this study, FELs of TEVp and mutants were constructed by utilizing radius of gyration (Rg) and RMSD value with different colors indicating different energies. The conformation with lower energy was represented in pale yellow, indicating greater stability compared to other simulated conformations (darker green). The thermodynamic stability of proteins is represented by the depth of energy minima, while the kinetic stability of proteins is represented by the height of potential barriers between energy minima. The associated free-energy landscape revealing that the WT enzyme could display four major conformational states, while eTEV exhibited two transition states, indicating they had the dispersed conformational states. However, uTEV3 exhibited a clear groove with the lowest energy, indicating a strong binding to the substrate and occupying a stable conformation.

### 2.2. Investigation of Binding Pocket Dynamics and Interactions

**Figure 5:**
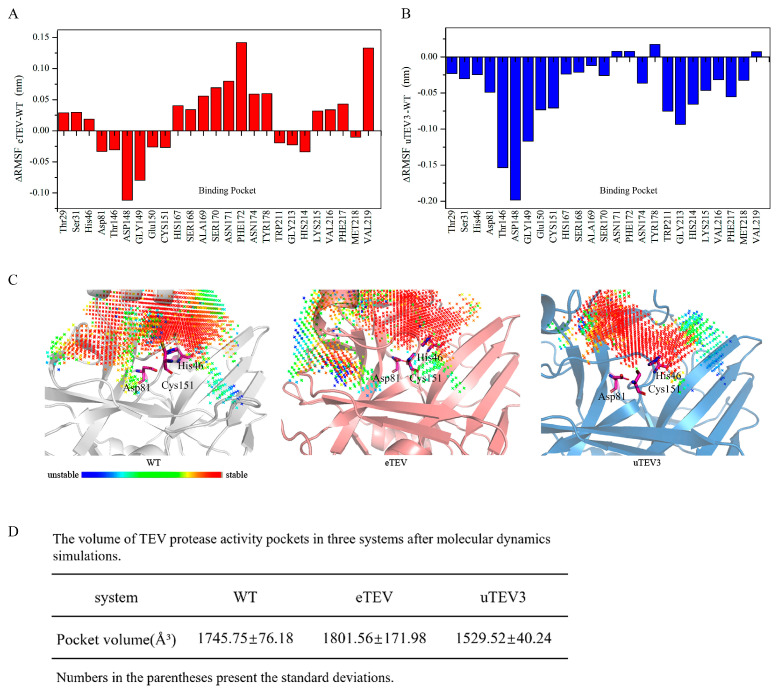
Pocket change analysis of WT and the two variants. (**A**,**B**). Flexible differences in the active sites between mutants and WT. (**C**) Stability of pockets in WT, eTEV, and uTEV3. (**D**) The volume of activity pockets in three systems during the last 10 ns of MD simulations (Website of Protein-Plus: https://proteins.plus/, accessed on 26 January 2024) [19].

In this study, we investigated the substrate-binding pocket dynamics and conformation of TEVp and its mutants. Comparison of the RMSF of the active site revealed that mutations had the opposite effects on the pocket for eTEV and uTEV3 (Figure 5A,B). The RMSF value of most pocket residues in eTEV was increased, indicating that the flexibility of the active site was enhanced. This might be beneficial for access to the substrate and the release of products. On the contrary, the RMSF value of most pocket residues in uTEV3 was decreased, suggesting the rigidity of the active pocket was increased, which might be conducive to the combination of binding pocket and substrate. Analysis of protein pocket dynamics by D3Pockets [18] is displayed in Figure 5C. The pockets detected during the MD process were composed of grid points. The color change in these points, from red to blue, represents the frequency at which they were observed in the MD trajectory. The red dot indicates a higher frequency of appearance in the pocket, while the blue dot indicates a lower frequency of appearance. Therefore, the area composed of red dots shows higher stability. Compared to WT, the proportion of red grids in the uTEV3 pocket was higher, indicating the higher stability of the active site, while eTEV exhibited flexibility in some areas of the pocket. Furthermore, the volumes of the active pocket of TEV protease and its two variants in molecular dynamics simulations were calculated. As shown in Figure 5D, the binding pocket volume of eTEV became larger (eTEV pocket volume = 1801.56 ± 171.98 Å). On the contrary, the active pocket volume of uTEV3 decreased (uTEV3 pocket volume = 1529.52 ± 40.24 Å) (Figure 5D).

**Figure 6:**
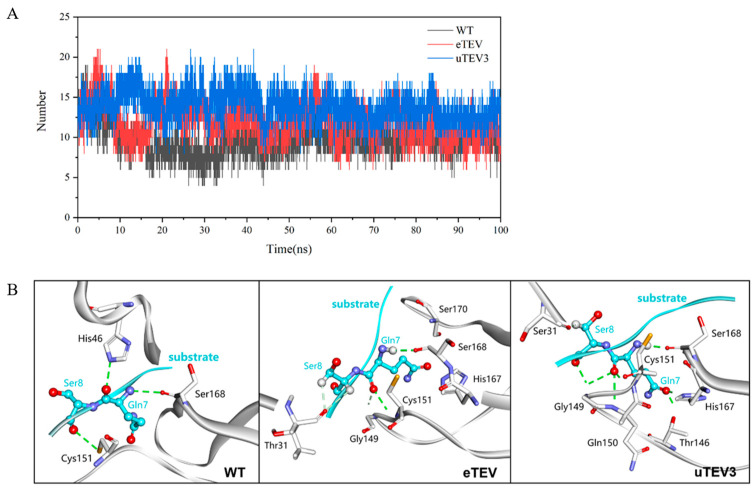
Hydrogen bonding analysis between TEV protease and substrate. (**A**) The hydrogen bonding between the TEV protease active pocket and the substrate peptide (ENLYFQSG) in three systems during molecular dynamics simulation changes over time. (**B**) H-bonds between TEV protease and substrate (Q-S). The substrate was represented in blue, the protease was represented in gray.

The stabilization of the substrate within the protein pocket comprises an extensive hydrogen-bond network. Our results showed that the number of hydrogen bonds formed between uTEV3 and the substrate was higher than that between eTEV and WT, indicating that uTEV3 had a higher affinity for the substrate than eTEV, which was conducive to the binding and reaction of the substrate at the active center (Figure 6A). Interactions (hydrogen bonds and hydrophobic interactions) between TEVps and peptide substrates were compared in Table 2. uTEV3 formed more hydrogen bonds with the substrate, while eTEV had fewer hydrogen bonds with the substrate. However, it was worth noting that uTEV3 and eTEV formed significantly more hydrogen bonds with the amino acids at the substrate hydrolysis site (Gln7↓Ser8). Figure 6B displays the interactions within the catalysis site (peptide Gln7-Ser8). In uTEV3, Thr146, Gly149, Gln150, Cys151, His167, and Ser168 formed hydrogen-bonding interactions with Gln7 of the substrate, and Ser31 and Gly149 contacted with substrate Ser8 by hydrogen bonds. In eTEV, it was found that Gly149, Cys151, His167, Ser168, Ser170 make hydrogen bonds with Gln7 of the peptide. And the mutated Thr31 in eTEV formed hydrogen bonds with Ser8. His46 and Ser168 in the active pocket had hydrogen-bonding interactions with Gln7, while Cys151 and Ser8 had hydrogen-bonding interactions in WT. The increase in hydrogen bonds between the substrate cleavage site (Gln7-Ser8) and variants of TEVp might contribute to the stabilization of the catalytic center and reaction intermediates, thereby facilitating the catalytic reaction.

Table 3 provides the substrate-binding free energies of TEVp and two mutants calculated using the MM-PBSA method. The results showed that uTEV3 had the strongest affinity with substrate, while there was no significant difference between WT and eTEV. This result was consistent with the reported enzyme reaction Michaelis constant (Km)of two variants. The Km of uTEV3 was significantly smaller than that of WT and eTEV, indicating a higher affinity between uTEV3 and the substrate, while WT and eTEV have similar Km values.

### 2.3. Residue Network and Community Analysis

To explore how mutations outside the substrate-binding pocket influence enzyme function, we analyzed the residue interaction network of TEVp–substrate complexes. This analysis aimed to understand structural communication and obtain the shortest path for each residue pair using the webPSN platform (http://webpsn.hpc.unimo.it/wpsn3.php, accessed on 26 January 2024) [20].

**Figure 7:**
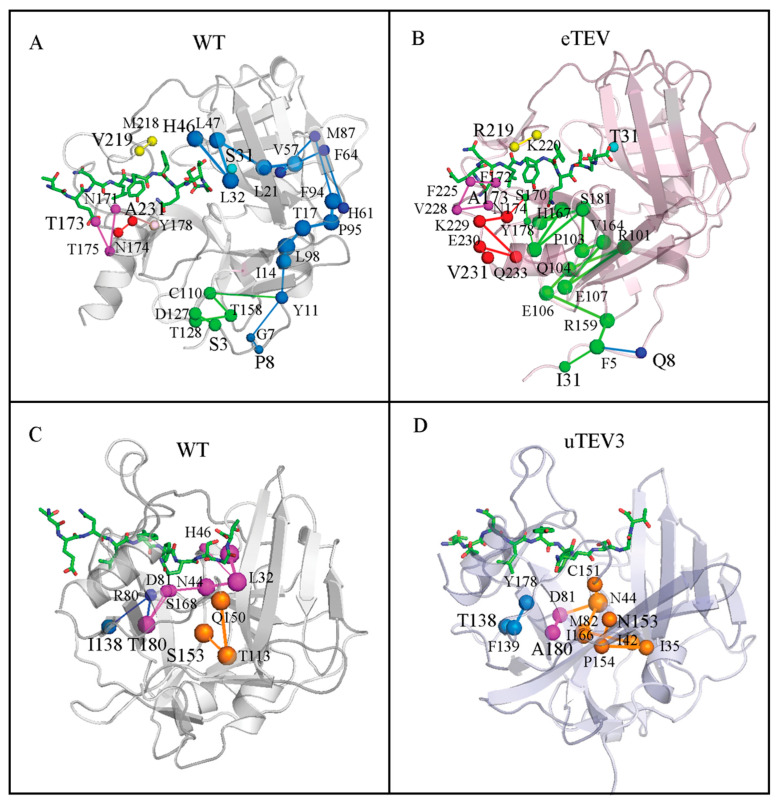
The communication pathway from mutation sites to the substrate-binding pocket of TEVp and mutants. (**A**) The communication pathway of WT corresponding to eTEV mutation sites. (**B**) The communication pathway of the mutated residues of eTEV. (**C**) The shortest communication pathway of WT corresponding to uTEV3 mutation sites. (**D**) Shortest communication pathway of the mutated residues of uTEV3.

Figure 7 analyzed the changes in the shortest interaction path between mutation sites and the substrate-binding pockets of WT, eTEV, and uTEV3. The data showed that those mutations alter the communication paths of protein structures.

In WT, the communication of the remote S3 was mediated by the D127-T128-T158-C110 pathway with the joining of the P8 pathway at Y11. Then, through a series of amino acids such as I14-L98-T17-P95-F94-M87-V57-L21-L47-L32-H46, it ultimately reached the catalytic H46 in the active center (Figure 7A). However, S3I and P8Q mutations in eTEV resulted in a significant shift in the interaction pathway. I3 and Q8 converge at F5 and ultimately affect substrate binding to the important residues H167, S170, and Y178 through a new path (Figure 7B). Similarly, the mutation residues 173 and 231 of eTEV outside the binding pocket also altered the interaction path. The same situation can also be observed in uTEV3 (Figure 7C,D). In uTEV3, S153 was replaced by Asn. The path of N153 to the active site was significantly prolonged. N153 affected catalytic residues C151 and D81 through P154-I35-I42-M82-I166-N44. We observed that N44 seemed to be an important node in both WT and uTEV3. In WT, N44 linked with catalytic amino acids H46 and D81, while in uTEV3, N44 interacted with catalytic amino acids D81 and C151. We will conduct more in-depth research in our future work.

## 3. Discussion

TEVp is able to cut specific substrate sequences, making it a valuable tool for studying protein function and interactions. Its applications have spanned various fields, including protein purification, protein interaction studies, protein variant generation, and therapeutic agent development [3]. However, its relatively slow catalytic rate poses a notable limitation [12,14,16,17,21,22]. To date, some successful examples mainly focus on directed evolution. Van den Berg et al. successfully produced the T17S,N68D,I77V variant through random mutagenesis, effectively improving the solubility and yield of the TEV protease [12]. The L56V,S135G variant was introduced by Cabrita LD et al., improving protein solubility and thermal stability [14]. A combination of mutations TEVp5M (T17S, L56V, N68D, I77V, and S135G) [23] identified by rational design [14] and high-throughput screening [12], exhibited the highest solubility and slightly elevated catalytic activity in vivo. Fan et al. found the TEVp5M-E106G variant enhanced the soluble production and cleavage activity of TEVp constructs [21]. The TEV-EAV variant (G79E,T173A) was introduced by YESS of combinatorial libraries [22], which retained high catalytic turnover. Subsequently, YESS 2.0, a highly versatile version of the yeast endoplasmic sequestration screening (YESS) system, was used to improve the TEV-EAV variant, which obtain eTEV variant (S3I, P8Q, S31T, T173A,V219R, and A231V) with a 2.25-fold higher catalytic efficiency, derived almost entirely from an increase in Kcat [16]. The uTEV3 variant (I138T, S153N, and T180A) was produced using a yeast-based platform for directed evolution of protease catalytic properties, in which catalytic activity had also been significantly improved [17]. Despite these successes, there is often no clear rationale as to why certain mutations lead to improvements, especially those far from the active site [24,25]. A detailed structure–function analysis is required to pinpoint the molecular mechanisms responsible for the observed enhancements.

In this study, we have selected two promising mutants, eTEV [16] and uTEV3 [17], for molecular dynamics simulations along with WT to investigate the relationship between mutations and the structural function. These two variants exhibit contrasting mechanisms for modulating enzyme performance. Specifically, eTEV preserves its Km value but significantly boosts Kcat, thereby enhancing catalytic efficiency without altering substrate affinity. Conversely, uTEV3 maintains a stable Kcat but escalates overall enzyme activity by effectively lowering its Km, thus improving substrate-binding affinity (Table 1).

Our results revealed that mutations in the variants had different effects on the structure mainly in terms of flexibility. Essentially, these changes altered the dynamic properties of the enzymes and had different effects on their functional efficiency. Our molecular dynamics simulations showed that eTEV exhibited a higher RMSF compared to the WT enzyme, which was indicative of increased flexibility in its structure (Figure 2C). The enhanced flexibility of eTEV resulted in its binding pocket possessing more dynamic properties (Figure 5A,C) and the enhanced mobility could be a contributing factor to the observed increase in Kcat without affecting Km, suggesting an improved ability for the protein to transition between conformations during the catalytic cycle. In contrast, the mutations in uTEV3 resulted in a more compact and stable active site pocket for substrate binding (Figure 5B,C). This suggests that while eTEV gains improved catalytic activity through the increased flexibility of its binding pocket, uTEV3 achieves enhanced enzyme efficiency by tightening the interaction with its substrate (Table 2) and reducing non-productive conformations (Figure 4), thereby lowering Km without affecting Kcat. The stabilization of the active site in uTEV3 could lead to better substrate recognition and binding, ultimately boosting its overall enzymatic performance. We also noticed that regardless of how the flexibility of the active pocket changes, the catalytic triad His46, Asp81, and Cys151 of eTEV, and uTEV3 retained their stability, ensuring that core enzymatic activity was not compromised (Figure 2). Moreover, both variants showed increased interactions between the enzyme and substrate at the cleavage site (Table 2). This suggests that preserving the functional core and strategically enhancing substrate catalytic site interactions are crucial for improving enzyme efficiency and specificity in engineering enzymes.

The mutation sites of eTEV are predominantly found in the N-terminal and C-terminal regions of the protein sequence (Figure 1). Notably, with the exception of Thr31 and Arg219, which may have implications for catalytic activity due to their proximity to the active site pocket, the remaining mutant residues do not directly occupy the active site region, suggesting their influence on enzyme function might be exerted through affecting the protein’s structure or dynamics in a way that optimizes substrate/product access and/or release without altering the catalytic machinery itself. In the case of uTEV3, the mutant residues, I138T, S153N, and T180A, are also not located within the direct confines of the substrate-binding pocket. This implies that these mutations exert their influence on enzyme activity through an indirect mechanism, possibly by stabilizing interactions outside the active site region that may allosterically modulate the pocket’s conformation or accessibility.

Therefore, we mapped the communication pathways between mutant residues and the pocket (Figure 7) of the TEVps. In the study of proteins, analyzing residue networks and communities can help in exploring the interaction patterns within proteins and how mutations affect their structure and functions. Residue networks can identify the "pathway" from mutated residues to the active site. These pathways may directly relate to the interaction between the mutated residue and the substrate-binding region, or indirectly transmit the influence through other residues [26,27,28].

The mutations of eTEV and uTEV3 extensively influence the structure, especially the S3I and P8Q of eTEV showed different connection point network characteristics compared with WT (Figure 7). In addition, we found some reported mutated residues associated with enzyme activity on these pathways, such as T17 [14] and E106 [21]. The role of residues on these communication pathways will be further explored in our future research.

In summary, the MD simulation described herein demonstrates how the activity of TEV proteases is influenced by mutations that are remote from the active site. Mutations outside the active site residues could affect the dynamic movement of the binding pocket by altering residue networks and communication pathways, thereby having a profound impact on reactivity. This work empowers us to anticipate and logically interpret the impacts of mutations, thereby deepening our comprehension of protein functionality and expediting protein engineering endeavors aimed at optimizing TEVp activities or devising innovative functionalities. Our team is currently working in this direction.

## 4. Materials and Methods

### 4.1. Structure Preparation

At present, the structure of the full-length TEVp has not yet been elucidated. Therefore, we built the structure of full-length TEVp WT (without substrate) using the Robetta server (Comparative Modeling) developed by the Baker lab (http://robetta.bakerlab.org/, accessed on 26 January 2024) [29,30]. Robetta is a protein structure prediction service containing relatively fast and accurate deep learning-based methods, RoseTTAFold and TrRosetta. Full-length TEVp WT–substrate complexes were constructed using MOE2022.02 software based on this model and the X-ray structure of the truncated TEV protease–substrate complex (PBD ID: 1LVB). The full-length eTEV and uTEV3–substrate complexes were mutated and constructed on the basis of full-length TEVp WT–substrate complexes. And then, full-length TEVp (WT, eTEV, and uTEV3)–substrate complexes were used to perform molecular dynamics simulations for a duration of 100 ns. Three initial structures of MD simulations are given in the Supplementary Materials.

### 4.2. Molecular Dynamic Simulation

All molecular dynamics (MD) simulations were performed using the GROMACS 2018.8 package [31], along with the standard CHARMM 27 force field [32]. To replicate the protein’s aqueous environment, the TIP3P water model was employed, and protein molecules were subjected to hydrogenation using the GROMACS 2018.8 package’s pdb2gmx module. Additionally, one Na^+^ ion and three Cl^-^ ions were introduced to neutralize the system and render it electrically balanced [33].

To initiate the process, we used the steepest descent method and Verlet integrator for 10,000 steps to reduce the maximum force less than 1000 kJ·mol^−1^·nm^−1^ in order to minimize energy and rectify atom-level interactions. Subsequently, simulations were carried out under conditions of 300 K temperature and 1 atm atmospheric pressure, involving constraint equilibrium calculations spanning 200 ps NVT (number of molecules, volume, and temperature) and 1 ns NPT (number of molecules, pressure, and temperature). Temperature coupling was accomplished using the v-rescale algorithm with a coupling constant of 0.1 ps on the two groups (the protein and the solvent and ion) separately. To handle long-range electrostatic interactions, the Particle Mesh Ewald (PME) algorithm was employed, the cut-off values for the van der Waals interactions was 1.2nm and the truncation threshold was 1.2 nm. Pressure calculation was facilitated using the Parrinello–Rahman algorithm, utilizing a coupling constant of 0.5 ps.

In summary, a dynamic simulation was conducted for a duration of 100 ns with a time step of 2 fs. The system was maintained at a temperature of 300 K and a pressure of 1 atm. Output data were recorded at intervals of 10 ps. This procedure was repeated two times to generate 6 groups of simulations, each consisting of 100 ns of independent simulation performed. A diverse array of analyses was performed on the MD simulation trajectories for each complex system. These analyses included the evaluation of parameters such as root mean square deviation (RMSD), root mean square fluctuation (RMSF), radius of gyration (Rg), and the characterization of the free-energy landscape.

The interpretation of results derived from the molecular dynamics simulations was enriched through the utilization of various analytical tools including GROMACS 2018.8, Discovery Studio 2019 Client, Pymol, and additional software applications and online platforms. The free-energy topography was drawn using sham commands of GROMACS 2018.8 and used Python to view the images. This comprehensive toolkit facilitated the in-depth analysis and interpretation of the intricate simulation outcomes.

### 4.3. Binding Energy Calculations

To calculate the binding free energy and interaction between TEV protease and its substrate, molecular mechanics Poisson–Boltzmann surface area (MM-PBSA) was used [34] based on 1000 frames of trajectory with an interval of 1 frame. Without considering the entropy term, the calculation value became the effective binding free energy (ΔG_bind_), which was calculated using Equation (1):

ΔG_bind_ = ΔG_gas_ + ΔG_sol_ (1)

where the ΔG_gas_ is the molecular mechanical energy in the gas phase and the ΔG_sol_ is the solvation energy. The process was covered through the thermodynamic cycle. Then, the ΔG_gas_ of TEVp–substrate complexes could be further calculated using Equation (2):

ΔG_gas_ = ΔE_bonded_ + ΔE_non-bonded_ = (ΔE_bond_ + ΔE_angle_ + ΔE_dihedral_) + (ΔE_ele_ + ΔE_vdW_) (2)

where the ΔE_bonded_ includes the molecular internal energies: ΔE_bond_, ΔE_angle_, and ΔE_dihedral_. And the non-bonded interaction ΔE_non-bonded_ is composed of electrostatic (ΔE_ele_) and vdW (ΔE_vdW_) interactions. Since the dynamic process does not involve the breaking or formation of intramolecular bonds, the ΔG_gas_ can also be expressed as the sum of ΔE_ele_ and ΔE_vdW_. Then, the solvation energy was calculated using Equation (3):

ΔG_sol_ = ΔG_polar_ + ΔG_nonpolar_ (3)

where ΔG_polar_ is the electrostatic or polar components to the solvation free energy evaluated by the Poisson–Boltzmann (PB) model, and ΔG_nonpolar_ is the hydrophobic or nonpolar components proportional to the molecular solvent accessible surface area (SASA).

## Figures and Tables

**Table 1:** Kinetic parameters for wild-type and mutant TEV proteases ^1^.

Enzyme	Mutation Sites	Km (mM)	Kcat (s^−1^)	Kcat/Km (mM^−1^s^−1^)
WT	—	0.069 ± 0.03	0.16 ± 0.02	2.23 ± 1.02 [16]
eTEV	S3I, P8Q, S31T, T173A, V219R, A231V	0.065 ± 0.012	0.41 ± 0.02	6.31 ± 1.2 [16]
uTEV3	I138T, S153N, T180A	0.022 ± 4	0.15 ± 0.01	6.82 [17]

^1^ With the peptide substrate ENLYFQSG.

**Table 2:** Interactions between the TEVps and the peptide substrate.

	WT (Residue)– Peptide (Residue)	eTEV (Residue)– Peptide (Residue)	uTEV3 (Residue)– Peptide (Residue)
Hydrogen Bonds	Lys141-Glu2	His214-Glu2	His214-Glu2
	Tyr178-Glu2	Ser170-Leu4	Lys215-Glu2
	His214-Glu2	Lys215-Leu4	Asn171-Asn3
	Asn171-Asn3	Phe217-Leu4	Phe172-Asn3
	Phe172-Asn3	Ala169-Tyr5	Lys215-Asn3
	Ser170-Leu4	Ser170-Tyr5	Ser170-Leu4
	Lys215-Leu4	Asn174-Tyr5	Lys215-Leu4
	Val216-Leu4	Phe217-Tyr5	Val216-Leu4
	Phe217-Leu4	Ser168-Phe6	Phe217-Leu4
	Ala169-Tyr5	Phe217-Phe6	Ala169-Tyr5
	Ser170-Tyr5	Gly149-Gln7 *	Ser170-Tyr5
	Asn174-Tyr5	Cys151-Gln7 *	Asn174-Tyr5
	Phe217-Tyr5	His167-Gln7 *	Phe217-Tyr5
	Ser168-Phe6	Ser168-Gln7 *	Phe217-Phe6
	Phe217-Phe6	Ser170-Gln7 *	Ser168-Phe6
	His46-Gln7 *	Thr31-Ser8 *	Thr146-Gln7 *
	Ser168-Gln7 *	Thr31-Gly9	Gly149-Gln7 *
	Cys151-Ser8 *	Gly149-Gly9	Gln150-Gln7 *
	Ser31-Gly9		Cys151-Gln7 *
	Gly149-Gly9		His167-Gln7 *
			Ser168-Gln7 *
			Ser31-Ser8 *
			Gly149-Ser8 *
			Ser31-Gly9
Hydrophobic Interactions	Ala169-Leu4	His214-Glu2	Ala169-Leu4
	Tyr178-Leu4	Ala169-Leu4	Tyr178-Leu4
	His214-Leu4	Tyr178-Leu4	Val216-Leu4
	Val216-Leu4	Val216-Leu4	Lys220-Tyr5
	Lys220-Tyr5	Lys220-Tyr5	Phe225-Tyr5
	His46-Phe6	His46-Phe6	His46-Phe6
	Val216-Phe6	Ala169-Phe6	Ala169-Phe6
		Val216-Phe6	Val216-Phe6

* The scissile site of the substrate: Gln7 (P1)–Ser8 (P1′).

**Table 3:** MM-PBSA energy analysis of proteases bound to substrates, in kcal/mol.

System	WT	eTEV	uTEV3
ΔG_gas_	−255.39 ± 2.29	−267.71 ± 0.86	−240.72 ± 0.59
ΔG_solv_	188.58 ± 1.70	202.19 ± 0.79	169.44 ± 0.57
Δtotal	−66.81 ± 0.63	−65.51 ± 0.24	−71.28 ± 0.20

Numbers in parentheses present the standard deviations.

## Data Availability

The data presented in this study are available within the article.

## Supplementary Materials

The following supporting information can be downloaded at: https://www.mdpi.com/article/10.3390/molecules29051071/s1.
